# Distinct effects of calorie restriction on adipose tissue cytokine and angiogenesis profiles in obese and lean mice

**DOI:** 10.1186/1743-7075-9-64

**Published:** 2012-06-29

**Authors:** Eveliina Kurki, Jin Shi, Essi Martonen, Piet Finckenberg, Eero Mervaala

**Affiliations:** 1 Institute of Biomedicine, Pharmacology, Biomedicum Helsinki, University of Helsinki, P.O.Box 63, FI-00014, Helsinki, Finland

**Keywords:** adipose tissue, cytokines, angiogenesis, calorie restriction

## Abstract

**Background:**

Obesity associates with low-grade inflammation and adipose tissue remodeling. Using sensitive high-throughput protein arrays we here investigated adipose tissue cytokine and angiogenesis-related protein profiles from obese and lean mice, and in particular, the influence of calorie restriction (CR).

**Methods:**

Tissue samples from visceral fat were harvested from obese mice fed with a high-fat diet (60% of energy), lean controls receiving low-fat control diet as well as from obese and lean mice kept under CR (energy intake 70% of *ad libitum* intake) for 50 days. Protein profiles were analyzed using mouse cytokine and angiogenesis protein array kits.

**Results:**

In obese and lean mice, CR was associated with 11.3% and 15.6% reductions in body weight, as well as with 4.0% and 4.6% reductions in body fat percentage, respectively. Obesity induced adipose tissue cytokine expressions, the most highly upregulated cytokines being IL-1ra, IL-2, IL-16, MCP-1, MIG, RANTES, C5a, sICAM-1 and TIMP-1. CR increased sICAM-1 and TIMP-1 expression both in obese and lean mice. Overall, CR showed distinct effects on cytokine expressions; in obese mice CR largely decreased but in lean mice increased adipose tissue cytokine expressions. Obesity was also associated with increased expressions of angiogenesis-related proteins, in particular, angiogenin, endoglin, endostatin, endothelin-1, IGFBP-3, leptin, MMP-3, PAI-1, TIMP-4, CXCL16, platelet factor 4, DPPIV and coagulation factor III. CR increased endoglin, endostatin and platelet factor 4 expressions, and decreased IGFBP-3, NOV, MMP-9, CXCL16 and osteopontin expressions both in obese and lean mice. Interestingly, in obese mice, CR decreased leptin and TIMP-4 expressions, whereas in lean mice their expressions were increased. CR decreased MMP-3 and PAI-1 only in obese mice, whereas CR decreased FGF acidic, FGF basic and coagulation factor III, and increased angiogenin and DPPIV expression only in lean mice.

**Conclusions:**

CR exerts distinct effects on adipocyte cytokine and angiogenesis profiles in obese and lean mice. Our study also underscores the importance of angiogenesis-related proteins and cytokines in adipose tissue remodeling and development of obesity.

## Background

Adipose tissue expansion during positive energy balance is characterized by adipocyte hypertrophy and visceral adipose tissue accumulation [1,2]. These pathogenic anatomic abnormalities in adipose tissue may trigger metabolic and immune responses that promote obesity-linked disorders, such as type 2 diabetes, hypertension, dyslipidemia and vascular diseases [1,2].

The normal physiological function of white adipose tissue (WAT) is to store the excess energy as neutral triglycerides, from which stored energy can be released for use at other sites to ensure continuous availability of energy despite highly variable energy supplies [3]. In addition, adipose tissue is an active endocrine organ that communicates with many other organs through the production of various secretory proteins, hormones and cytokines that are collectively referred as adipokines having both pro- and anti-inflammatory activities [4]. Obesity induces adipose tissue dysfunction, shifting it toward production of pro-inflammatory adipokines and infiltration of macrophages, which eventually leads to the development of chronic low-grade inflammation [4,5]. This obesity-induced inflammatory state contributes to systemic metabolic dysfunction that associates with obesity-linked disorders [4,6].

The unique characteristic of adipose tissue is it plasticity as it continuously undergoes expansion and regression throughout adult life. Adipose tissue is highly vascularized organ and therefore the expansion of adipose tissue requires continuous remodeling of the vascular network [7-9]. The finding that adipose tissue explants induces blood vessel formation [10], and in turn adipose tissue endothelial cells promote preadipocytes differentiation [11], proves that adipogenesis is linked to angiogenesis. Furthermore, adipose tissue explants have been utilized clinically to promote wound healing [12], indicating the strong angiogenic activity of adipose tissue.

The concomitant occurrence of adipogenesis and angiogenesis suggests that modulation of angiogenesis may impair adipose tissue development, and thus it could offer a novel therapeutic option for the treatment of obesity [13]. Anti-angiogenesis agents, including the small chemical compound TNP-470 and broad-spectrum of endogenous protein inhibitors, angiostatin and endostatin, have been shown to prevent genetically and diet-induced obesity in animals [14,15]. The better understanding the regulation of pro- and anti-angiogenic components during adipogenesis, might provide new targets and approaches for the treatment of obesity and related metabolic disorders.

In contrast to the excess calorie intake and subsequent obesity, calorie restriction (CR) effectively ameliorates the incidence of obesity and related metabolic disorders [16]. In this study high-fat diet fed C57Bl/6 J mice were used as model of diet-induced obesity, and cytokine and angiogenesis-related protein profiles were studied between obese and lean mice using antibody array systems. In addition, we performed 30% CR for obese and lean mice and investigated how CR affects protein profiles, and whether the effects are different between obese and lean mice.

## Materials and methods

### Animals and metabolic measurements

Six-week-old male C57Bl/6 J mice were purchased from Charles River Europe (Sulzfeld, Germany). The animals were housed 5 per cage in a standard experimental animal laboratory, illuminated from 07.00 to 19.00 h (temperature 22 ± 1°C). The protocols were approved by the Animal Experimentation Committee of the University of Helsinki, Finland and the principles of laboratory animal care (NIH publication no. 85–23, revised 1985) were followed. The mice had free access to tap water during the experiment. After a 1-week acclimatization period, the mice (n = 6) were fed a high-fat diet (60% of energy from fat, D05031101M, Research Diets Inc., New Brunswick, NJ, USA) *ad libitum* for 100 days to induce obesity. Lean mice (n = 6) were fed a normal rodent diet (Harlan Tekland 2018, Harlan Holding, Inc, Wilmington, DE, USA) *ad libitum* for 100 days. After 100 days, obese and lean mice were maintained under calorie restriction (CR, 70% energy of *ad libitum* energy intake) for 50 days. Obese (n = 7) and lean (n = 6) controls were fed a same high-fat diet (D05031101M) and normal rodent diet *ad libitum*, respectively, during the whole experiment for 150 days.

The food consumption was monitored daily and the body weight once per week by using a standard table scale (Ohaus Scour™ Pro, SP4001, Nänikon, Switzerland). The energy intake was calculated based on the food consumption and nutritional data. The body fat content was analyzed by dual-energy x-ray absorptiometry (DEXA, Lunar PIXImus, GE Healthcare, Chalfont St. Giles, UK) before and after CR.

For oral glucose tolerance test (OGTT), mice were fasted 6 h and after that glucose were given by gavage (1 g/kg glucose, 20% glucose solution). Blood glucose was determined with a glucose metre (Super Glucocard™ II, GT-1630, Arkray Factory Inc., Shiga, Japan) on blood samples taken from the tail vein at time points 0, 15, 30, 60 and 90 min after the gavage. Areas under the curve (AUC) (blood glucose x time) were calculated.

After the treatment period, the mice were housed in metabolic cages for 24 h and faeces samples were collected. The faeces were weighted and stored at −70°C until assayed. The faecal fat content was determined by Schmid-Bondzynski-Ratzlaff method. The apparent fat digestibility (%) was determined from fat intake and faecal fat content as described previously [17], using the formula: the apparent fat digestibility (%) = 100 × [(fat intake − fecal fat content)/fat intake].

At the end of the experiment, the mice were rendered unconscious with CO_2_/O_2_ (95:5% v/v; AGA, Riihimäki, Finland) and decapitated. The abdominal fat pads were removed, washed with saline, blotted dried and weighted.

### Adipocyte size

Adipocyte cross sectional area was performed as described in detail elsewhere [18]. Briefly, the fat pads were fixed in 10% formalin and embedded in paraffin with routine techniques. Sections (4 μm) of paraffin-embedded adipose tissue samples were cut with a microtome and mounted on charged glass, deparaffinized in xylene and stained. The adipocyte cross-sectional area was determined under a conventional light microscope (x10 magnification) in a “blinded” fashion in four fields from each sample (n = 6-7/group) by Leica QWin Standard-software (Leica Microsystems Imaging Solutions Ltd, Cambridge, UK).

### Cytokine and angiogenesis protein analyses

Proteins from abdominal fats (n = 3/group) were isolated with PBS containing complete protease inhibitors (Roche Diagnostics, Neuilly-Sur-Seine, France). Fat samples were homogenized using a Bertin Precellys 24 homogenizer (Bertin Technologies, Aix en Provence, France), ceramic beads (Precellys CK14, Bertin Technologies), and a protocol consisting of 5000 rpm for 20s repeated twice. Homogenized samples containing Triton® X-100 (Sigma, St. Louis, Mo., USA) with a final concentration of 1% were frozen at −70°C overnight and centrifuged 10,000 g for 5 min. Protein analysis was performed using mouse cytokine array panel A (represents 40 mouse cytokine proteins) and mouse angiogenesis array kits (represents 53 mouse angiogenesis related proteins) (Proteome Profiler™ antibody arrays, R&D Systems, Inc., MN, USA) according to the protocol of the manufacturer. Proteins in the 3 samples (250 μg/sample) from each group were pooled together and 750 μg of the total protein was used for one membrane. Chemiluminescence solution (ECLplus; Amersham Biosciences, GE Healthcare, Little Chalfont, UK) was used for protein detection. The protein expression in membranes was visualized by FLA-9000 fluorescent image analyzer (Fujifilm, Tokyo, Japan). Proteins were spotted in duplicates on membranes, and the relative protein expression between samples was determined by analyzing the pixel densities of spots in each arrays.

### Statistical analysis

Data are presented as means ± SEM. Statistically significant differences in mean values were tested by ANOVA followed by the Newman-Keuls multiple comparison test. p values <0.05 were considered statistically significant. GraphPad Prism, version 4.02 (GraphPad Software, Inc., San Diego, Calif., USA), was used for the statistical analyses.

## Results

### Mice characterization and changes in glucose tolerance and apparent fat digestibility

The daily energy intake did not differ between obese and lean mice (Table 1). The energy intake of calorie restricted obese and lean mice was approximately 70% of *ad libitum* intake as stated in study plan. The body weight of obese mice was 1.4-fold higher than in lean mice (Table 1). The increase in body weight correlated with 2.7-fold increase in body fat percentage, whereas no difference was seen in lean body mass between obese and lean mice (Table 1). CR in obese mice decreased body weight 11.3%, and in lean mice CR led to 15.6% reduction in body weight. In obese mice, the body weight loss correlated with 4.0% reduction in body fat percentage and 8.9% reduction in lean body mass. Corresponding values for lean mice were 4.6% reduction in body fat percentage and 10.1% reduction in lean body mass.

**Table 1:** Daily energy intake, body weight, body fat percentage, lean body mass, area under the curve (AUC) of blood glucose and apparent fat digestibility of obese and lean mice and mice kept under calorie restriction (CR)

	**Obese**	**Obese CR**	**Lean**	**Lean CR**	**N**	**ANOVA p-value**
Daily energy intake (kJ/mouse/day)	61.36 ± 3.40	42.59 ± 0.44^a,b^	58.60 ± 5.16	37.23 ± 0.22^a,b^	3	p=0.0013
Body weight (g)	42.11 ± 1.40^a^	37.36 ± 0.61^a,b^	29.74 ± 0.65	25.11 ± 0.82^a,b^	12-14	p<0.001
Body fat (%)	38.54 ± 1.28^a^	34.54 ± 1.68^a,b^	14.17 ± 0.88	9.61 ± 0.39^a,b^	12-14	p<0.001
Lean body mass (g)	26.47 ± 0.66	24.12 ± 0.44^b^	25.66 ± 0.63	23.06 ± 0.66^a,b^	12-14	p=0.0009
AUC blood glucose (mmol*min/ml)	1259.17 ± 147.91^a^	1074.67 ± 22.88^a^	722.72 ± 29.30	608.78 ± 27.97^b^	6	p<0.001
Apparent fat digestibility (%)	90.66 ± 5.46^a^	93.81 ± 2.06^a^	65.12 ± 2.54	76.54 ± 3.21^a,b^	6-7	p<0.001

Data is presented as mean ± SEM.

^a^ P < 0.05 compared to lean.

^b^ P < 0.05 compared to obese.

Oral glucose tolerance was higher in lean mice than in obese, but CR did not affect oral glucose tolerance (Table 1). The apparent fat digestibility was increased in obese mice compared to lean mice, and CR in lean mice increased apparent fat digestibility, whereas no significant change was seen in obese mice (Table 1).

### Adipocyte size

**Figure 1:**
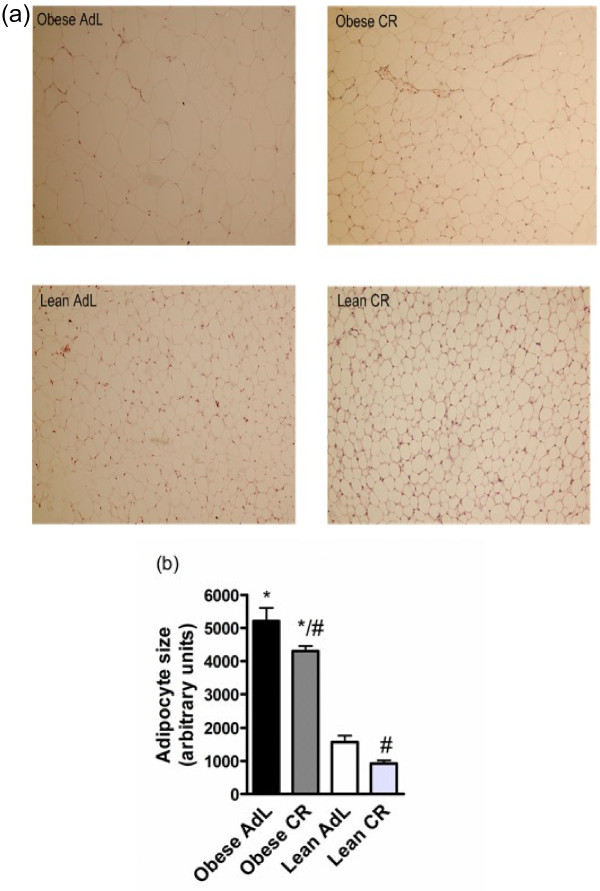
**Representative photomicrographs (Original magnification × 100) of adipoctytes (A) and adipocyte size shown in histogram (B, n = 6−7).** Data are presented as mean ± SEM. * denotes the significant (p < 0.05) difference in comparison with the lean group, # denotes the significant (p < 0.05) difference in comparison with the obese group.

The adipocyte size, measured as adipocyte cross-sectional area, was significantly higher in obese mice than in lean mice (Figure 1A, C). Compared to *ad libitum* fed counterparts, CR in obese mice significantly decreased adipocyte size, and it tended to decrease in lean mice, but the difference did not reach statistical significance.

### Adipose tissue cytokine protein profile

Mouse cytokine array kit was used to analyze the protein expression of 40 different pro- and anti-inflammatory cytokines in adipose tissue. Two cytokines IL-12 p70 ja MIP-1α were not detected in any study group, and eotaxin was detected only in calorie restricted lean mice ( Additional file 1: Table S1).

**Figure 2:**
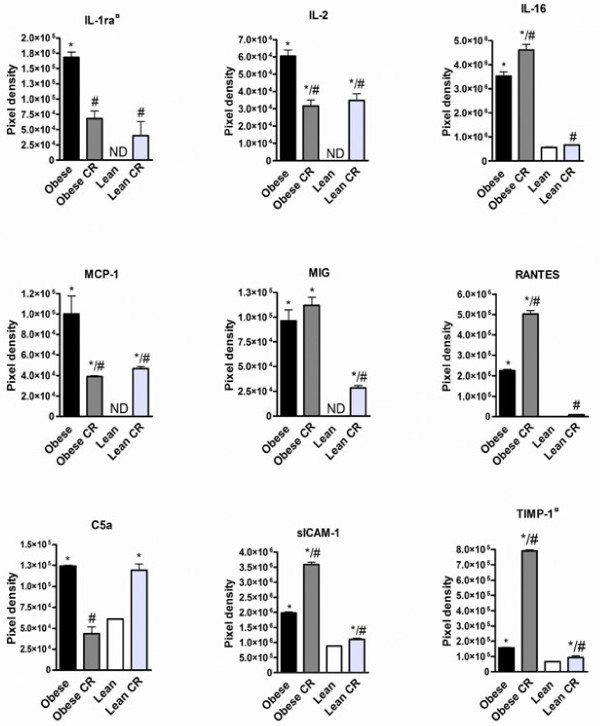
** Protein expression of cytokines in adipose tissue of *ad libitum* fed or calorie restricted (CR) obese and lean mice.** ¤ indicates the anti-inflammatory cytokines. * denotes the significant (p < 0.05) difference in comparison with the lean group, # denotes the significant (p < 0.05) difference in comparison with the obese group.

Diet-induced obesity induced cytokine protein expression, and together 27 cytokines were expressed at higher level in obese mice as compared to lean controls ( Additional file 1: Table S1). The highly expressed proteins included interleukins IL-1ra, IL-2 and IL-16, chemokines MCP-1, MIG and RANTES, complement component C5a, adhesion molecule sICAM-1 and matrix matrix metallopeptidase inhibitor TIMP-1 (Figure 2).

Cytokine protein profiling revealed that CR in obese mice decreased the protein expression of 22 proteins and increased 5 proteins expression ( Additional file 1: Table S1). CR when performed for lean mice showed opposite effect, and the protein expression of 26 proteins was increased by CR compared to *ad libitum* fed lean mice ( Additional file 1: Table S1). Comparison between caloric restricted mice and *ad libitum* fed counterparts revealed that CR highly in obese mice and moderately in lean mice increased sICAM-1 and TIMP-1 expression (Figure 2). CR uniquely in obese mice increased IL-16 and RANTES protein expression and decreased IL-1ra protein expression (Figure 2). In addition, CR uniquely in lean mice increased MIG protein expression (Figure 2). Several CR induced changes were distinct between obese and lean mice, and CR in obese tended to decrease and lean mice increase IL-2, MCP-1 and C5a protein expression (Figure 2).

### Adipose tissue angiogenesis protein profiles

Mouse angiogenesis array kit was used to analyze the protein expression of 53 pro- or anti-angiogenesis proteins in adipose tissue. All proteins were detectable at least in one study group.

**Figure 3:**
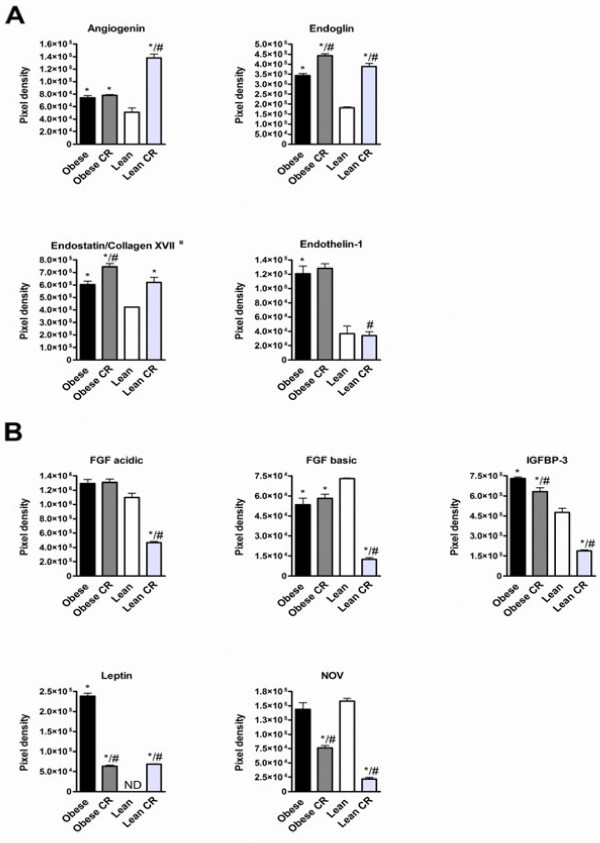
**Protein expression of angiogenic growth factors (A) and cell growth regulators (B) in adipose tissue of *ad libitum* fed or calorie restricted (CR) obese and lean mice.** ¤ indicates the anti-angiogenic proteins. * denotes the significant (p < 0.05) difference in comparison with the lean group, # denotes the significant (p < 0.05) difference in comparison with the obese group.

**Figure 4:**
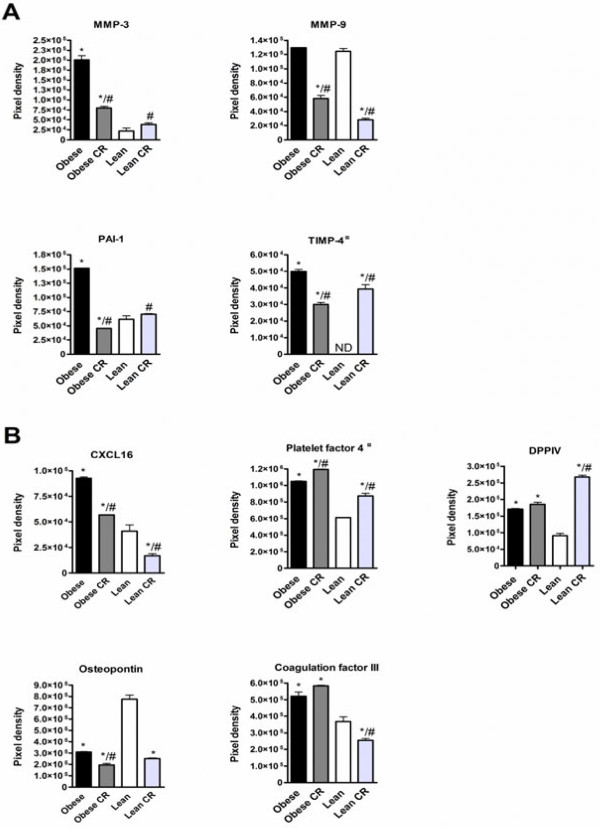
** Protein expression of proteases and protease inhibitors (A), and chemokines and cell adhesion proteins (B) in adipose tissue of *ad libitum* fed or calorie restricted (CR) obese and lean mice.** ¤ indicates the anti-angiogenic proteins. * denotes the significant (p < 0.05) difference in comparison with the lean group, # denotes the significant (p<0.05) difference in comparison with the obese group.

17 proteins were expressed at higher level and 6 proteins at lower level in obese mice adipose tissue compared to lean mice ( Additional file 2: Table S2). The protein expression of cell growth regulators angiogenin, endoglin, endostatin and endothelin-1 were increased in obese mice adipose tissue compared to lean mice (Figure 3A). In addition, the protein expression of angiogenic growth factors IGFBP-3 and leptin were increased, and FGF basic was decreased in obese mice compared to lean mice (Figure 3B). Proteases modulate extracellular matrix and they have important role in initiation of angiogenesis [19]. The protein expression of protease MMP-3 and protease inhibitors PAI-1 and TIMP-4 were increased in obese mice compared to lean mice (Figure 4A). Furthemore, chemokines CXCL16 and platelet factor 4, adhesion molecule DPPIV and coagulation factor III were higher expressed in obese than in lean mice, whereas osteopontin was lower expressed in obese mice than in lean mice (Figure 4B).

Comparison of calorie restricted obese mice with *ad libitum* fed obese controls showed that 14 proteins were expressed at lower and 6 proteins at higher level ( Additional file 2: Table S2). In lean mice, CR caused major differences, and the expression of 32 proteins were increased and the level of 9 proteins were decreased compared to *ad libitum* fed lean mice ( Additional file 2: Table S2). 12 of the highly expressed proteins were detected only in lean CR group. Cell growth regulators endoglin and endostatin/collagen XVII were increased by CR both in obese and lean mice (Figure 3A). Angiogenin was uniquely increased by CR in lean mice (Figure 3A). CR both in obese and lean mice decreased angiogenic growth factors IGFBP-3 and NOV protein expression (Figure 3B). Furthermore, CR uniquely in lean mice decreased FGF acidic and FGF basic protein expression (Figure 3B). CR had opposite effect on leptin expression by decreasing leptin expression in obese mice and increasing expression in lean mice to the level found in calorie restricted obese mice (Figure 3B).

Proteases were regulated in response to body weight changes and CR both in obese and lean mice decreased protease MMP-9 protein expression compared to *ad libitum* fed mice (Figure 4A). CR uniquely in obese mice decreased MMP-3 and PAI-1 protein expression (Figure 4A). The protein expression of TIMP-4 was decreased by CR in obese mice, while in lean mice CR increased expression (Figure 4A). In addition, CR both in obese and lean mice decreased CXCL16 and osteopontin expression and increased platelet factor 4 expression (Figure 4B). CR uniquely in lean mice increased DPPIV protein expression, and decreased coagulation factor III protein expression compared to *ad libitum* fed lean mice (Figure 4B).

## Discussion

Accumulating evidence suggests an important role for low-grade inflammation and adipose tissue remodeling in the development of obesity. In the present study we investigated the adipose tissue cytokine and angiogenesis-related protein profiles from obese and lean mice by using sensitive high-throughput protein arrays. Furthermore, we examined the influence of calorie restriction (CR) on adipose tissue protein profiles. The important finding from the present study was that obesity is associated with simultaneous induction of several cytokines and angiogenesis-related proteins in adipose tissue. CR decreased body weight and body fat percentage to a similar extent in obese and lean mice. However, CR showed opposite effects on protein profiles between obese and lean mice. CR largely ameliorated cytokine and angiogenesis-related protein expression in obese mice, while in lean mice marked upregulation of several proteins was seen.

Accumulating evidence suggests a close relationship between the amount of visceral fat, metabolic disturbances and cardiovascular diseases [4,6]. Adipose tissue dysfunction leads abnormal cytokine secretion thus inducing the development of low-grade inflammatory state that contributes to obesity-linked metabolic disorders such as type 2 diabetes [4,6]. To study further the molecular mechanisms mediating adipose tissue inflammation in obesity, we characterized the cytokine expression profiles from visceral fat. We were able to demonstrate that obesity is associated with up-regulation of several pro-inflammatory cytokines, including IL-1ra, IL-2, IL-16, MCP-1, MIG, RANTES, C5a and sICAM-1. It is of great interest that CR in obese mice markedly attenuated cytokine overexpression, whereas in lean mice CR actually increased the levels of most of the above mentioned pro-inflammatory cytokines in the adipose tissue. Distinct effects of CR on cytokine expression profiles in obese and lean mice can not been explained by differences in the study design as both the body weight and body fat percentage were decreased by CR to a similar extent in obese and lean mice. Our findings are in good agreement with the study by Fenton et al. [20] demonstrating that CR increases serum cytokine levels in lean mice. Our findings are also consistent with the recent report by Wang et al. [21] showing that CR ameliorates adipose tissue inflammation in diet-induced obese mice, in particular when CR is carried out by restricting intake of HFD. Further studies are thus warranted to investigate the cellular mechanisms mediating the opposite effects of CR on adipose tissue inflammatory response between obese and lean mice.

Adipose tissue is the highly vascularised tissue, and fat mass expansion is closely linked to angiogenesis [7,8]. Although the cellular mechanisms regulating adipose tissue-related angiogenesis are still poorly understood, several pro- and anti-angiogenic components have been identified [7]. As adipose tissue angiogenesis is known to be critical for adipogenesis, a more deep understanding of the regulation of adipose tissue angiogenesis may provide novel drug targets for obesity and obesity-related disorders. We therefore examined the expression of 53 different pro- and anti-angiogenic factors in adipose tissue. We were able to demonstrate that obesity is associated with marked alterations in the protein expression of cell growth regulators, angiogenic growth factors and proteases as well as their inhibitors. The present study also revealed that CR has a pronounced modulating effect on adipose tissue protein expression profiles. However, inclusive nature of our angiogenic findings should be underlined; we did not perform histological analyses to characterize the vasculature, endothelial cells or ECM proteins in adipose tissue. Further studies are thus warranted to investigate how the altered adipose tissue protein expression profiles influence the vasculature. Furthermore, as obesity has been shown to alter collagen and elastin expression in adipose tissue [22], it would be important to examine the influence of CR on collagen metabolism in future.

Our study showed that leptin was one of the angiogenic growth factor that is highly sensitive to body weight changes. Leptin is an adipocyte-derived hormone that regulates food intake and energy homeostasis. Leptin is also a potent angiogenic factor. Leptin induces angiogenesis through activation of its own receptor in endothelial cells leading to activation of Stat3 pathway and enhancement of its DNA-binding activity [23]. Leptin also indirectly activates angiogenesis by up-regulating VEGF mRNA expression via activation of the Jak/Stat3 signaling pathway [24]. In addition, leptin has a synergistic effect with FGF basic (also called FGF-2) and VEGF on stimulation of new blood vessel formation [25]. In the present study, leptin was high expressed in obese mice compared to lean mice. Interestingly, higher protein expression of leptin in obese mice associated with lower expression of FGF basic, but there was trend toward increased in PlGF-2 (VEGF homologue) and VEGF-B (see in supplemental data) protein expression between obese and lean mice. In obese mice CR down-regulated leptin expression and up-regulated VEGF expression. In lean mice the effect of CR on leptin expression was opposite; CR up-regulated leptin expression, down-regulated FGF basic and up-regulated VEGF expression. These findings indicate distinct effects of CR on adipose tissue leptin expression between obese and lean mice and suggest also interaction between leptin, FGF basic and VEGF family members.

In the present study angiogenic growth factors endostatin and endoglin were up-regulated by CR both in obese and lean mice. Endostatin is an endogenous angiogenesis inhibitor [26], and treatment with endostatin reduces body weight of obese mice [14]. Silha et al. showed recently that plasma levels of vascular growth factors as well as the angiogenesis inhibitor endostatin are increased in obese individuals [27]. Endoglin in turn is a membrane glycoprotein that serves as a receptor for members of the TGF-β superfamily proteins. It is highly expressed on proliferating vascular endothelial cells and it has crucial role in vascular development and disease [28]. However, the effects of endoglin on adipose tissue remodeling in obesity are still elusive. In the present study we demonstrated that endothelin-1 level in the adipose tissue was increased in obese mice. Previous studies have revealed that endothelin-1 induces insulin resistance by suppressing glucose uptake [29,30] and lipolysis in adipocytes [31-34] through ET_A_ receptors. Increased plasma endothelin-1 levels have also been reported in obese subjects with metabolic syndrome [35]. However, the present study revealed that CR does not reduce adipose tissue endothelin-1 levels.

Pericellular proteases have been shown to play an important role in regulating angiogenesis. Proteases participate in extracellular matrix (ECM) remodeling and in angiogenic processes by generating pro- and anti-angiogenic factors from ECM proteins and by processing growth factors and receptors [19]. Plasminogen activator-plasmin system (fibrinolytic system) and matrix metalloproteinases (MMPs) are two major component of proteolytic system [19]. Plasminogen activator inhibitor-1 (PAI-1, also known as serpine E1) is an inhibitor of fibrinolytic system exerting several physiological and pathophysiologial effects related to tumorigenesis, inflammation, thrombosis and metabolic disturbances such as obesity and insulin resistance [36]. Data from studies investigating the effects of PAI-1 on adipogenesis are controversial; some studies using a diet-induced obese mouse models suggest that PAI-1 deficiency has little if any effect on the development of obesity [37,38], while other studies report prevention of obesity and insulin resistance in mice lacking PAI-1 [39]. Furthermore, PAI-1 inhibitor tiplaxtinin has been shown to prevent adipogenesis and diet-induced obesity [40,41]. In the present study PAI-1 expression correlated with body weight, and significantly higher PAI-1 expression were found in obese mice. We also noticed that CR down-regulated PAI-1 expression only in obese mice. Our findings thus suggest an important role for PAI-1 in the development of adipose tissue.

The expression of matrix metallopeptidases (MMPs) in the adipose tissue were also altered in diet-induced obese mice. We report here increased MMP-3 expression in obese mice and down-regulation of MMP-3 in the adipose tissue by CR. It is of great interest that CR down-regulated MMP-9 expression both in obese and lean mice, although no difference was detected when the mice were fed *ad libitum*. Up-regulation of MMP-3 and down-regulation of MMP-9 mRNA expression have been reported recently in expanding adipose tissue [42]. Enhanced adipose tissue development and increased adipose tissue blood vessel density have been demonstrated in MMP-3 deficient mice kept on high-fat diet [43]. Moreover, MMPs inhibitors have been shown to inhibit angiogenesis and to reduce body weight in diet-induced obese mice [14,44-46].

MMPs are inhibited by endogenous tissue inhibitors (TIMPs), and we here demonstrated upregulation of tissue inhibitors of metalloproteinases TIMP-1 and TIMP-4 with obesity. CR increased TIMP-1 expression both in obese and lean mice, whereas TIMP-4 expression was down-regulated by CR in obese mice and up-regulated in lean mice. TIMP-1 deficient mice has been shown to gain less weight and develop less adipose tissue when fed with high-fat diet and this was related to lower leptin levels detected in TIMP-1 deficient mice [47]. These findings suggest an important role for proteolytic system in adipose tissue development during diet-induced obesity and during weight reduction induced by CR.

Recent studies suggest an important role for osteopontin in the development of HFD-induced insulin resistance and, regulation of vascular and adipose tissue inflammation [48,49]. Weight loss has been shown to decrease plasma osteopontin levels [50]. We also demonstrated that CR decreased adipose tissue osteopontin expression both in obese and lean mice. Surprisingly, in contrast to some previous studies [50,51], we were unable to demonstrate obesity-induced osteopontin overexpression in the adipose tissue. Finally, we here reported increased expression of CXCL16 in obese mice. Furthermore, we were able to demonstrate that CR decreased adipose tissue CXCL16 expression both in lean and obese mice. Previous studies have linked CXCL16 and its receptor CXCR6 to inflammation-associated cancers [52], renal fibrosis [53], and vascular inflammatory diseases, such as atherosclerosis [54]. Further studies are warranted to investigate the role of CXCL16/CXCR6 axis in adipose tissue remodeling.

## Conclusion

Using diet-induced obese mice as experimental model of obesity we here demonstrate that obesity is associated with induction of several cytokines and angiogenesis-related proteins in the adipose tissue. Although calorie restriction decreased body weight and body fat percentage to a similar extent in obese and lean mice, the influence of CR on adipose tissue protein profiles was largely opposite; whereas CR ameliorated cytokine and angiogenesis-related protein expression in obese mice, we noticed an upregulation of several proteins by CR in lean mice. These findings support the notion of modulating adipose tissue cytokines and/or angiogenesis-related proteins to ameliorate the development of obesity. The present study also suggests that CR might exert detrimental effects on adipose tissue remodeling in lean mice.

## Competing interests

The authors have no competing interest.

## Authors’ contribution

JS and EK conducted the study. EK and EM did the cytokine and angiogenesis protein arrays. EK analyzed the dataset and wrote the manuscript. All authors read and approved the final manuscript.

## Supplementary Material

Additional file 1 **Table S1.**Pixel densities (mean ± SEM) of cytokines in each study group. Click here for file

Additional file 2 **Table S2.** Pixel densities (mean ± SEM) of cytokines in each study group. Click here for file
