# Supplementary material for: Distinct effects of calorie restriction on adipose tissue cytokine and angiogenesis profiles in obese and lean mice
Source: Nutr Metab (Lond). 2012 Jun 29;9:64. doi: 10.1186/1743-7075-9-64 (PMC3478179; doi:10.1186/1743-7075-9-64)
Supplement: Additional file 1 — Table S1.Pixel densities (mean ± SEM) of cytokines in each study group. [file 1743-7075-9-64-S1.doc]

Supplemental table 1. Pixel densities (mean ± SEM) of cytokines in each study group.

|  | **Obese** | **Obese CR** | **Lean** | **Lean CR** | **Classification** |
| --- | --- | --- | --- | --- | --- |
| **BCL** | 109864.25 ± 6942.56^a^ | 18735.85 ± 0.00^d^ | 13257.00 ± 0.00 | 94486.76 ±12674.55^a^ | Chemokine |
| **C5a** | 123971.38 ± 1009.92^a^ | 43770.85 ± 8047.31^d^ | 60911.78 ± 166.81 | 119436.20 ±7420.64^a^ | Complement component |
| **G-CSF** | 35125.99 ± 600.57^a^ | ND^d^ | ND | 28362.07 ± 882.08^a^ | Growth factor |
| **GM-CSF** | 28874.30 ± 2589.24^a^ | ND^d^ | ND | 47563.24 ± 2204.01^a^ | Growth factor |
| **I-309** | 35305.49 ± 2192.38^a^ | 100632.24 ± 0.00^a/c^ | ND | 53724.51 ± 32.97^a/c^ | Chemokine |
| **Eotaxin** | ND | ND | ND | 5702.25 ± 2372.51 | Chemokine |
| **sICAM-1** | 1978513.89 ± 33299.99^a^ | 3587252.05 ± 79277.81^a^ | 874600.95 ± 16125.25 | 1101060.49 ± 39359.93^a/d^ | Adhesion molecule |
| **IFN-γ** | 10641.46 ± 507.48^a^ | ND^d^ | ND | 20535.80 ± 3162.67^a^ | Interferon |
| **IL-1α** | 53789.94 ± 11305.25^a^ | 9676.24 ± 0.00^d^ | ND | 52860.94 ± 2837.37^a^ | Interleukin |
| **IL-1β** | 33130.99 ± 265.08^a^ | ND^d^ | ND | 5118.84 ± 134.96^a^ | Interleukin |
| **IL-1ra^¤^** | 168431.20 ± 8688.34^a^ | 68165.55 ± 12647.70^d^ | ND | 40002.36 ± 23237.48^d^ | Interleukin |
| **IL-2** | 60430.65 ± 3509.74^a^ | 31573.85 ± 3553.31^a/d^ | ND | 34827.58 ± 3857.06^a/d^ | Interleukin |
| **IL-3** | 77227.77 ± 20385.72 | 22976.85 ± 0.00 | 24268.55 ± 0.00 | 83885.02 ± 15225.62 | Interleukin |
| **IL-4^¤^** | 26293.83 ± 1377.36^a^ | ND^d^ | 5949.81 ± 0.00 | 51612.77 ± 6022.88^a^ | Interleukin |
| **IL-5^¤^** | 23179.79 ± 721.39^a^ | ND^d^ | ND | 19016.59 ± 197.81^a/d^ | Interleukin |
| **IL-6** | 18003.56 ± 2840.46^a^ | ND^d^ | ND | 18574.05 ± 256.20^a^ | Interleukin |
| **IL-7** | 29980.39 ± 15432.27 | ND | ND | 37387.72 ± 1135.25 | Interleukin |
| **IL-10^¤^** | 2804.92 ± 1450.12 | ND | ND | 32788.75 ± 3950.58^a/c^ | Interleukin |
| **IL-13** | 10809.45 ± 0.00 | ND | ND | 18963.42 ± 2941.16^a/c^ | Interleukin |
| **IL-12 p70** | ND | ND | ND | ND | Interleukin |
| **IL-16** | 3535699.74 ± 158033.29^a^ | 4609388.05 ± 231204.19^a/c^ | 571389.56 ± 15566.93 | 667141.66 ± 9834.31^d^ | Interleukin |
| **IL-17** | 38811.12 ± 6081.78^a^ | 7217.54 ± 0.00^d^ | ND | 14619.60 ± 3715.57^d^ | Interleukin |
| **IL-23** | 26506.26 ± 589.94^a^ | ND^d^ | ND | 16056.23 ± 449.73^a/d^ | Interleukin |
| **IL-27** | 22484.48 ± 769.72^a^ | 12775.16 ± 0.00^a/d^ | ND | 13147.01 ± 1626.39^a/d^ | Interleukin |

|  | **Obese** | **Obese CR** | **Lean** | **Lean CR** | **Classification** |
| --- | --- | --- | --- | --- | --- |
| **IP-10** | 87121.48 ± 41273.24 | 71201.77 ± 45247.77 | 59591.99 ± 31902.75 | 120640.29 ± 24772.49 | Interleukin |
| **I-TAC** | 18265.85 ± 6503.19 | 4894.32 ± 0.00 | 11463.59 ± 739.35 | 55172.29 ± 7545.55^a/c^ | Chemokine |
| **KC** | 41636.78 ± 4417.03^a^ | 5272.54 ± 0.00^d^ | 12180.47 ± 0.00 | 43849.43 ± 42.27^a^ | Chemokine |
| **M-CSF** | 58683.94 ± 580.45^a^ | 34844.55 ± 2579.78^a/d^ | ND | 57271.93 ± 44.65^a^ | Growth factor |
| **MCP-1** | 100124.72 ± 17559.96^a^ | 39191.77 ± 699.23^a/d^ | ND | 46523.43 ± 1848.60^a/d^ | Chemokine |
| **MCP-5** | 14878.78 ± 2716.02 | 5974.05 ± 3835.28 | ND | 31234.10 ± 4186.04^a/c^ | Chemokine |
| **MIG** | 96229.46 ± 11153.26 | 112024.27 ± 8104.27^a/c^ | ND | 28651.32 ± 2328.20^a^ | Chemokine |
| **MIP-1α** | ND | ND | ND | ND | Chemokine |
| **MIP-1β** | 34999.19 ± 5922.82^a^ | 3310.00 ± 0.00^d^ | ND | 5539.24 ± 1409.77^d^ | Chemokine |
| **MIP-2** | 18137.74 ± 1270.14^a^ | ND^d^ | ND | 5483.95 ± 0.00^d^ | Chemokine |
| **RANTES** | 223576.71 ± 5939.31^a^ | 502637.27 ± 16911.73^a/c^ | ND | 8684.40 ± 1009.65^d^ | Chemokine |
| **SDF-1** | 3280.99 ± 0.00 | 10875.00 ± 0.00 | ND | 2089.25 ± 0.00 | Chemokine |
| **TARC** | 121156.51 ± 54496.47 | 12435.05 ± 49645.81 | 131798.46 ± 64424.57 | 135524.59 ± 33828.53 | Chemokine |
| **TIMP-1^¤^** | 156265.66 ± 2342.35^a^ | 791865.85 ± 6090.69^a/c^ | 66967.09 ± 926.56 | 93004.84 ± 11109.83^a/d^ | MMP inhibitor |
| **TNF-α** | 57670.62 ± 1138.30^a^ | 3510.85 ± 0.00^d^ | 17329.51 ± 0.00 | 48080.04 ± 490.36^a^ | Growth factor |
| **TREM-1** | 44612.21 ± 4206.64^a^ | 11780.85 ± 3710.31^a/d^ | ND | 42302.73 ± 1107.80^a^ | Receptor |

¤ indicates anti-inflammatory proteins.

a indicates that protein expression is significantly (p˂0.05) up-regulated compared to lean.

b indicates that protein expression is significantly (p˂0.05) down-regulated compared to lean.

c indicates that protein expression is significantly (p˂0.05) up-regulated compared to obese.

d indicates that protein expression is significantly (p˂0.05) down-regulated compared to obese.
