# Supplementary material for: Distinct effects of calorie restriction on adipose tissue cytokine and angiogenesis profiles in obese and lean mice
Source: Nutr Metab (Lond). 2012 Jun 29;9:64. doi: 10.1186/1743-7075-9-64 (PMC3478179; doi:10.1186/1743-7075-9-64)
Supplement: Additional file 2 — Table S2. Pixel densities (mean ± SEM) of cytokines in each study group. [file 1743-7075-9-64-S2.doc]

| Supplemental table 2. Pixel densities (mean ± SEM) of cytokines in each study group. | | | | | |
| --- | --- | --- | --- | --- | --- |
|  | **Obese** | **Obese CR** | **Lean** | **Lean CR** | **Classification** |
| **ADAMTS1^¤^** | ND | 9064.53 ± 0.00^a/c^ | ND | 21787.94 ± 885.74^a/c^ | Metallopeptidase activity |
| **Amphiregulin** | ND | 799.96 ± 0.00 | ND | 26621.37 ± 714.13^a/c^ | Growth factor |
| **Angiogenin** | 74042.75 ± 3654.54^a^ | 78226.04 ± 1083.35^a^ | 50980.02 ± 7174.00 | 138151.59 ± 599.81^a/c^ | Cell growth regulator |
| **Angiopoietin-1** | ND | ND | ND | 24179.96 ± 3173.70^a/c^ | Angiopoietins |
| **Angiopoietin-3** | ND | ND | ND | 11620.77 ± 2017.63^a/c^ | Angiopoietins |
| **Coagulation Factor III** | 520538.30 ± 25872.30^a^ | 583042.46 ± 4614.50^a^ | 367630.07 ± 29027.23 | 253835.58 ± 12468.09^b/d^ | Coagulation cascade |
| **CXCL16** | 92804.04 ± 1382. 74^a^ | 56968.46 ± 255.35^a/d^ | 40913.07 ± 5989.40 | 16633.77 ± 2002.73^b/d^ | Chemokine |
| **Cyr61** | ND | ND | ND | 32284.74 ± 905.13^a/c^ | Cell adhesion |
| **DLL4^¤^** | ND | ND | ND | 41983.57 ± 1057.62^a/c^ | Cell growth regulator |
| **DPPIV** | 171421.08 ± 2009.03^a^ | 185409.71 ± 6275.96^a^ | 90994.73 ± 6827.05 | 267558.34 ± 5690.24^a/c^ | Cell adhesion |
| **EGF** | ND | ND | ND | 14759.36 ± 128.88^a/c^ | Growth factor |
| **Endoglin** | 342994.99 ± 9649.46^a^ | 443348.71 ± 9320.04^a/c^ | 182783.59 ± 3321.44 | 388345.89 ± 15147.61^a/c^ | Cell growth regulator |
| **Endostatin/**  **Collagen XVIII^¤^** | 602657.74 ± 27288.24^a^ | 744688.17 ± 25685.97^a/c^ | 422045.76 ± 904.84 | 620095.23 ± 40627.81^a^ | Cell growth regulator |
| **Endothelin-1** | 120715.01 ± 10455.02^a^ | 128347.21 ± 6228.54^a^ | 36935.05 ± 10641.99 | 34080.65 ± 5289.79^d^ | Cell growth regulator |
| **FGF acidic** | 1294944.22 ± 53887.35 | 1307894.67 ± 47905.53 | 1098865.18 ± 59238.45 | 467509.10 ± 15966.62^b/d^ | Growth factor |
| **FGF basic** | 53297.24 ± 5081.98^b^ | 58223.21 ± 2933.46^b^ | 72914.18 ± 549.21 | 12406.47 ± 1350.33^b/d^ | Growth factor |
| **FGF-7** | ND | ND | 6603.92 ± 5516.20 | 38369.27 ± 1573.34^a/c^ | Growth factor |
| **Fractalkine** | ND | ND | ND | 34436.15 ± 850.24^a/c^ | Chemokine |
| **GM-CSF** | ND | ND | ND | 32938.32 ± 1197.44^a/c^ | Growth factor |
| **HB-EGF** | ND | ND | ND | 28732.73 ± 1598.51^a/c^ | Growth factor |
| **HGF** | ND | ND | ND | 20492.09 ± 947.52^a/c^ | Growth factor |
| **IGFBP-1** | ND | 1750.20 ± 0.00 | ND | 23951.16 ±2604.94^a/c^ | Growth factor |
| **IGFBP-2** | 155164.66 ± 4712.73 | 183659.21 ± 6619.53 | 139235.21 ± 18230.11 | 100393.37 ± 8949.55 | Growth factor |
| **IGFBP-3** | 731499.57 ± 9345.63^a^ | 632068.67 ± 30294.53^a/d^ | 476941.15 ± 30432.05 | 188435.89 ± 6829.99^b/d^ | Growth factor |
| **IL-1α** | 10688.62 ± 406.11 | ND^b/d^ | 10303.29 ± 469.24 | ND^b/d^ | Interleukin |
| **IL-1β** | 5493.12 ± 2543.88 | ND | 2096.09 ± 1062.22 | ND | Interleukin |
|  | **Obese** | **Obese CR** | **Lean** | **Lean CR** | **Classification** |
| **IL-10¤** | ND^b^ | ND^b^ | 31252.07 ± 2082.68 | 41288.67 ± 726.91^a/c^ | Interleukin |
| **IP-10^¤^** | ND^b^ | ND^b^ | 13706.56 ± 3930.39 | 32847.16 ± 1739.5^a/c^ | Chemokine |
| **KC** | ND | ND | ND | 19748.03 ± 491.9^a/c^ | Chemokine |
| **Leptin** | 238226.48 ± 6983.72^a^ | 63715.70 ± 2203.19^a/d^ | ND | 68481.10 ± 930.92^a/d^ | Growth factor |
| **MCP-1** | 21311.99 ± 2139.69^a^ | 6095.51 ± 262.69^a/d^ | ND | 27399.22 ± 710.93^a/c^ | Chemokine |
| **MIP-1α** | ND | ND | ND | 14448.44 ± 1972.33^a/c^ | Chemokine |
| **MMP-3 (pro and mature form)** | 200612.56 ± 10534.36^a^ | 80311.51 ± 4140.31^a/d^ | 22164.17 ± 7132.37 | 38248.51 ± 4219.50^d^ | Metallopeptidase activity |
| **MMP-8 (pro form)** | 27828.95 ± 14524.99 | 1898.51 ± 0.00 | 8147.96 ± 3030.94 | 8581.54 ± 320.90 | Metallopeptidase activity |
| **MMP-9 (pro and active form)** | 129797.40 ± 273.04 | 58025.51 ± 4367.69^b/d^ | 124696.85 ± 3834.46 | 27900.09 ± 2075.61^b/d^ | Metallopeptidase activity |
| **NOV** | 143876.95 ± 11422.37 | 76306.20 ± 3974.31^b/d^ | 158290.08 ± 4420.36 | 22048.50 ± 2329.56^b/d^ | Growth factor |
| **Osteopontin** | 309900.53 ± 3369.95^b^ | 195813.21 ± 14295.46^b/d^ | 775951.99 ± 35863.95 | 251971.55 ± 6254.85^b^ | Cell adhesion |
| **PD-ECGF** | ND | 1807.14 ± 0.00 | 100569.96 ± 70370.35 | 38096.75 ± 2919.12 | Growth factor |
| **PDGF-AA** | ND^b^ | 11281.71 ± 244.97 | 25384.94 ± 8922.40 | 78490.45 ± 1586.12^a/c^ | Growth factor |
| **PDGF-AB/PDGF-BB** | ND | 12304.67 ± 387.47^a/c^ | ND | 38547.16 ± 1235.22^a/c^ | Growth factor |
| **Pentraxin-3^¤^** | ND | ND | ND | 25470.11 ± 663.28^a/c^ | Chemokine |
| **Platelet Factor 4^¤^** | 1049065.07 ± 7046.66^a^ | 1192942.67 ± 5821.47^a/c^ | 613192.50 ± 422.73 | 872810.57 ± 31796.45^a/d^ | Chemokine |
| **PιGF-2** | 3299.11 ± 2077.02 | 7035.71 ± 851.97 | 18056.08 ± 4199.83 | 13093.35 ± 4254.93 | Growth factor |
| **Prolactin^¤^** | 5753.22 ± 0.00 | 761.14 ± 0.00 | 1905.42 ± 0.00 | 10698.13 ± 899.69^a/c^ | Cell growth regulator |
| **Proliferin** | 15908.36 ± 3252.65^a^ | ND^d^ | 7246.88 ± 1962.51 | 1687.86 ± 467.34^d^ | Cell growth regulator |
| **SDF-1** | ND^b^ | 2 0417.46 ± 4461.50^b^ | 72205.30 ± 9368.04 | 58798.88 ± 1994.54^c^ | Chemokine |
| **Serpin E1** | 151334.80 ± 190.40^a^ | 45319.04 ± 391.35^b/d^ | 61286.44 ± 6233.09 | 70320.97 ± 1098.65^d^ | Protease inhibitor |
| **Serpin F1^¤^** | 291172.75 ± 8975.61^a^ | 100041.96 ± 3020.00^a/d^ | 27143.88 ± 8000.35 | 60362.01 ± 2339.66^c^ | Protease inhibitor |
| **Thrombospondin-2^¤^** | ND | 5833.04 ± 613.36 | ND | 40456.55 ± 2723.68^a/c^ | Cell adhesion |
| **TIMP-1^¤^** | 53569.88 ± 3172.62^a^ | 37367.46 ± 2604.50^a/d^ | ND | 63419.33 ± 3165.80^a^ | Metallopeptidase activity |
| **TIMP-4^¤^** | 49899.70 ± 1328.03^a^ | 29927.54 ± 1281.85^a/d^ | ND | 39337.77 ± 2601.92^a/d^ | Metallopeptidase activity |
| **VEGF** | ND | 6584.96 ± 0.00^a/c^ | ND | 24800.74 ± 1114.92^a/c^ | Growth factor |
|  | **Obese** | **Obese CR** | **Lean** | **Lean CR** | **Classification** |
| **VEGF-B** | 17870.43 ± 1234.10 | 26291.54 ± 2633.15 | 32027.58 ± 4509.39 | 31033.16 ± 2579.79 | Growth factor |

¤ indicates anti-inflammatory proteins.

a indicates that protein expression is significantly (p˂0.05) up-regulated compared to lean.

b indicates that protein expression is significantly (p˂0.05) down-regulated compared to lean.

c indicates that protein expression is significantly (p˂0.05) up-regulated compared to obese.

d indicates that protein expression is significantly (p˂0.05) down-regulated compared to obese.
